# Wood Carbon Sponge/SiO_2_ Aerogel Composites with Anisotropic Broadband Microwave Absorption, Elastic Resilience, and Thermal Insulation

**DOI:** 10.1002/advs.77888

**Published:** 2026-09-21

**Authors:** Shuo Sun, Zhimeng Zhao, Weikai Zhan, Yonggang Jiang, Fengqi Liu, Yijie Hu, Junzong Feng, Jian Feng

**Affiliations:** ^1^ Science and Technology on Advanced Ceramic Fibers and Composites Laboratory, College of Aerospace Science and Engineering National University of Defense Technology Changsha People's Republic of China; ^2^ Hunan Provincial Key Laboratory of High‐Speed Aerodynamic Metamaterials Changsha People's Republic of China; ^3^ Department of Materials Science and Engineering, College of Aerospace Science and Engineering National University of Defense Technology Changsha People's Republic of China

**Keywords:** anisotropic architecture, compressible, electromagnetic wave absorption, SiO_2_ aerogel, thermal insulation, wood carbon sponge

## Abstract

Biomass‐derived carbon aerogels have attracted considerable interest for lightweight microwave‐absorption owing to their hierarchical porosity and renewability. While the structural anisotropy of natural wood is well recognized, its consequences for electromagnetic response remain largely unexplored. Herein, we utilize SiO_2_ aerogel‐incorporated wood‐derived carbon sponge as a model system to investigate its electromagnetic anisotropy in the axial, radial, and tangential directions. Governed by the aligned cellular architecture, a robust permittivity ordering (tangential > radial > axial) is revealed. Notably, the composite exhibits pronounced anisotropic absorption: the tangential direction achieves a minimum reflection loss of −64.1 dB, the radial direction delivers an ultra‐broadband effective absorption bandwidth of 11.6 GHz (covering the entire X and Ku bands) at a thickness of only 4.95 mm, and the axial direction also maintains strong absorption, with the overall performance surpassing most biomass‐derived carbon aerogels at comparable thicknesses. The composite also reveals a low thermal conductivity of ∼0.04 W/(m·K) and anisotropic compressibility. This work clarifies the intrinsic structure‐electromagnetic‐mechanical‐thermal correlations in anisotropic wood carbon sponges and offers a bioinspired paradigm for advanced lightweight stealth materials with concurrent thermal insulation.

## Graphical abstract

Anisotropic wood carbon sponge/SiO_2_ aerogel composites are constructed, revealing a robust direction‐dependent permittivity ordering (tangential > radial > axial). The radial direction delivers an ultra‐broadband effective absorption bandwidth of 11.6 GHz at only 4.95 mm, while the tangential direction offers superior compressibility and thermal insulation. This work turns the intrinsic anisotropy of natural wood into a practical design parameter for multifunctional stealth applications.

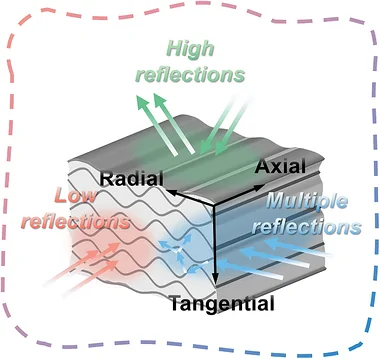

## 1 Introduction

Biomass‐derived carbon aerogels from wood [1, 2], bamboo [3, 4, 5], cotton [6, 7], and other plant tissues have emerged as promising candidates for lightweight microwave absorption owing to their intrinsic hierarchical porosity and renewable nature [8, 9, 10, 11, 12, 13]. Among these, balsa wood stands out as a particularly suitable precursor owing to its low density (∼0.1 g/cm^3^), high porosity, and well‐defined anatomical anisotropy. These features can be retained after carbonization to yield direction‐dependent electromagnetic properties that are less pronounced in other biomass‐derived carbons. While anisotropic microwave absorbers have been realized through ice‐templating and 3D printing, the intrinsic structural anisotropy of natural wood remains largely unexplored for direction‐dependent electromagnetic response. Existing studies on wood‐derived carbon absorbers are typically confined to measurements along a single direction [10, 14, 15], and the direction‐dependence of complex permittivity and microwave absorption remains poorly understood.

In wood anatomy, the three principal directions, which include axial (parallel to the tree growth direction), radial (parallel to the wood rays), and tangential (perpendicular to the wood rays), exhibit markedly distinct microstructural arrangements [16, 17, 18, 19, 20]. Specifically, the axial cross‐section reveals honeycomb‐like channels, the radial cross‐section exposes the extended planar surfaces of wood ray lamellae, and the tangential cross‐section presents a more homogeneous network punctuated by lenticular ray cross‐sections. Delignification, freeze‐drying, and carbonization convert native wood into a wood carbon sponge (WCS) while largely preserving this anisotropic cellular architecture. This directionally aligned pore network is expected to govern complex permittivity, impedance matching, and ultimately microwave absorption in a direction‐dependent manner. To experimentally establish this structure‐electromagnetic correlation, herein we select balsa wood as the precursor and fabricate a SiO_2_‐aerogel‐incorporated wood carbon sponge (WCS‐SiO_2_). The SiO_2_ aerogel serves a dual role: (i) acting as a low‐permittivity phase that moderates the excessive conductivity, particularly in the tangential direction, thereby improving impedance matching; (ii) introducing abundant carbon/SiO_2_ heterogeneous interfaces that serve as additional polarization centers, enhancing dielectric loss. This material design allows us to investigate the intrinsic anisotropic electromagnetic response of the carbon skeleton while simultaneously exploring the regulatory effect of a dielectric filler.

In this work, WCS and WCS‐SiO_2_ were characterized along the axial, radial, and tangential directions over 2–18 GHz. A robust permittivity ordering (tangential > radial > axial) was revealed, and the composite delivered an ultra‐broadband effective absorption bandwidth of 11.6 GHz in the radial direction and a minimum reflection loss of −64.1 dB in the tangential direction after SiO_2_ incorporation. The material also exhibited low thermal conductivity and enhanced anisotropic compressibility. These findings clarify the intrinsic structure–electromagnetic correlation in anisotropic wood‐derived carbon absorbers and offer a promising route toward multifunctional lightweight composites for stealth and thermal insulation applications.

## 2 Results and Discussion

### 2.1 Fabrication and Characterization

**FIGURE 1:**
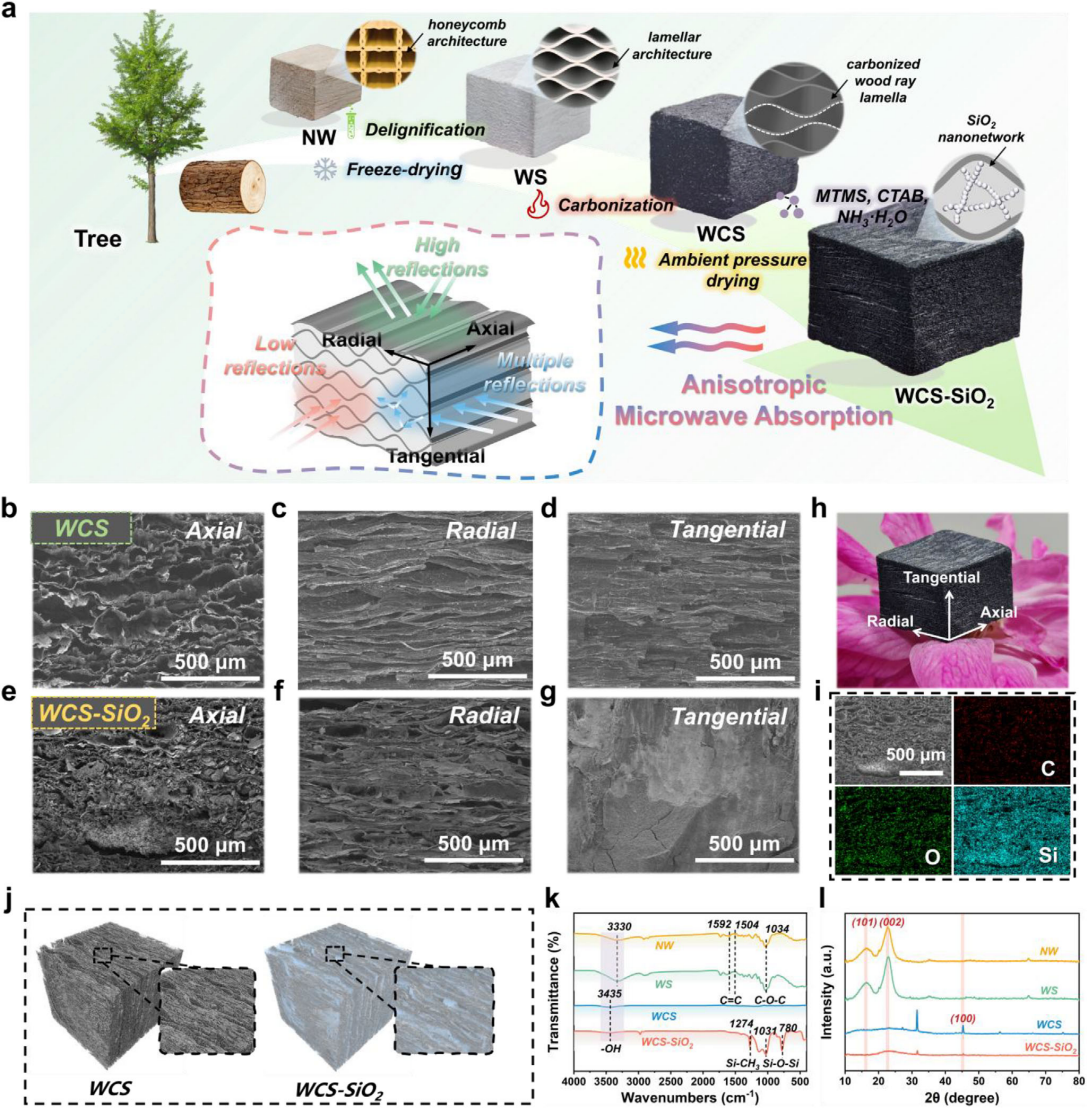
(a) Schematic illustration of the fabrication process of WCS‐SiO_2_. (b–d) SEM images of WCS viewed along the axial, radial, and tangential directions, respectively. (e–g) SEM images of WCS‐SiO_2_ viewed along the axial, radial, and tangential directions, respectively. (h) Optical photograph of WCS‐SiO_2_. (i) EDS elemental distribution maps of WCS‐SiO_2_, showing the homogeneous dispersion of C, O, and Si. (j) 3D micro‐CT analysis of WCS and WCS‐SiO_2_. (k) FT‐IR spectra, (l) XRD patterns of NW, WS, WCS, and WCS‐SiO_2_, respectively.

The fabrication process is schematically illustrated in Figure 1a. Natural wood (NW) was first subjected to delignification to remove lignin and partial hemicellulose, followed by freeze‐drying to obtain the wood sponge (WS) (Figure S1). The resulting WS was then carbonized under an argon atmosphere to yield the WCS. Subsequently, the obtained WCS was impregnated with a methyltrimethoxysilane (MTMS)‐derived organosilica sol. After vacuum‐assisted impregnation, gel aging, solvent exchange, and ambient pressure drying, the resulting composite (chemically a methylsilsesquioxane network) was finally obtained and is denoted as WCS‐SiO_2_ for brevity (Figure 1h). Figure S2 illustrates the density evolution of the samples across different preparation stages. After carbonization, the WCS exhibits a density of approximately 0.035 g/cm^3^, which increases to approximately 0.13 g/cm^3^ after SiO_2_ incorporation. Although the density increases upon incorporating SiO_2_, the integration of the SiO_2_ aerogel underpins the high thermal insulation, mechanical, and microwave absorption properties. Figure S2 presents the scanning electron microscopy (SEM) images of NW and WS, while Figure 1b–d displays the microstructures of WCS as viewed on the cross‐sections corresponding to the three principal anatomical directions. In the axial direction (the tree growth direction), the cross‐section reveals that natural wood exhibits a honeycomb cellular architecture partitioned by radially aligned wood rays (Figure S3). Following sequential delignification, freezing, and carbonization, the WCS develops a lamellar architecture composed of stacked, arch‐shaped layers (Figure 1b). The formation of this lamellar structure originates from the directional growth of ice crystals during freezing. Delignification ruptures the wood ray cells, enabling substantial water to accumulate within them. Owing to the higher water content in the wood rays, ice crystals preferentially nucleate and grow at these sites, leading to pronounced volume expansion [21]. The resultant expansion force compresses the adjacent wood fiber cells, driving them closer and densifying them until the honeycomb pores are fully closed. Moreover, the intrinsic non‐uniform thickness of individual wood rays causes uneven ice expansion, while a small number of vessels simultaneously exert opposing forces that locally restrict ice crystal growth. The interplay of these two factors ultimately produces the undulating lamellar structure. On the tangential cross‐section (perpendicular to the wood rays) (Figure 1c), the original honeycomb cellular architecture of the native wood is no longer discernible. Instead, the individual fiber cell walls have fused into a unified, interconnected carbon network, where the distinct lumina that once separated adjacent cells have largely disappeared. The structure appears as a continuous, undulating matrix, punctuated by the cross‐sectional profiles of the arch‐shaped layers and occasional residual micropores. On the radial cross‐section (parallel to the wood rays) (Figure 1d), the WCS displays the extended planar surfaces of the carbonized wood ray lamellae. These lamellae appear as continuous, dense carbon sheets oriented along the ray direction, with their large‐area faces fully exposed. The spaces between adjacent lamellae correspond to the broad interlayer voids (tens to hundreds of micrometers in width) formed by the directional ice‐crystal growth and subsequent removal during freeze‐drying. This pronounced structural anisotropy imparts the WCS with direction‐dependent architecture, laying the foundation for its anisotropic properties. After the incorporation of SiO_2_ aerogel, the original anisotropic architecture of the WCS is fully preserved, with SiO_2_ nanoparticles uniformly filling the interlayer voids of the carbon skeleton (Figure 1f–h). The EDS elemental distribution map (Figure 1i) reveals a homogeneous distribution of C, O, and Si elements, further confirming the uniform dispersion of the SiO_2_ aerogel throughout the WCS matrix. For brevity, the following nomenclature is adopted hereafter: WCS‐A, WCS‐T, and WCS‐R denote the axial, tangential, and radial directions of WCS, respectively, while WCS‐SiO_2_‐A, WCS‐SiO_2_‐T, and WCS‐SiO_2_‐R refer to the corresponding directions of the WCS‐SiO_2_ composites.

Three‐dimensional micro‐CT reconstruction (Figure 1j; Figure S4) further corroborates this observation, revealing a homogeneous distribution of the organosilica phase across the entire millimeter‐scale monolith without discernible aggregation or phase separation, thereby confirming that the vacuum‐assisted impregnation effectively delivers the sol into the interconnected pore network throughout the bulk material.

The pore structure of WCS and WCS‐SiO_2_ was characterized by nitrogen adsorption‐desorption measurements (BET) and mercury intrusion porosimetry (MIP) (Figure S5). The N_2_ adsorption‐desorption isotherm of WCS displays a type I profile with rapid uptake at low relative pressures and a nearly flat plateau, confirming a predominantly microporous structure formed by gas evolution during carbonization. The corresponding BJH pore size distribution shows a single peak at ∼2 nm. In contrast, WCS‐SiO_2_ exhibits a type IV isotherm with an H3 hysteresis loop, characteristic of a well‐developed mesoporous network. For WCS, the BJH analysis shows a single peak centered at approximately 2 nm, while MIP detects only macropores larger than 1000 nm, corresponding to the large interlayer voids. This indicates that the intrinsic porosity of the carbon skeleton is dominated by micropores formed during pyrolytic gas evolution. For WCS‐SiO_2,_ the BJH distribution extends broadly across 2–50 nm, and MIP reveals an additional mesopore population in the range of 10–100 nm, confirming that the organosilica aerogel introduces a well‐developed mesoporous network within the existing macroporous lamellar architecture.

The chemical characterization of the aerogels was carried out to elucidate their physicochemical property evolution throughout the reaction process. Figure 1k shows Fourier‐Transform infrared spectroscopy (FT‐IR) spectra of NW, WS, WCS, and WCS‐SiO_2_ over the range of 4000–400 cm^−1^. The characteristic lignin peaks at 1592 and 1504 cm^−1^ (aromatic skeletal vibrations) are significantly attenuated after delignification, and the characteristic cellulose peaks (O─H stretching at 3330 cm^−1^ and C─O stretching at 1034 cm^−1^) become pronounced, confirming the effective removal of lignin. After carbonization, the characteristic peaks of cellulose in WCS largely disappear, indicating the complete thermal decomposition of the polysaccharide framework and its conversion into carbon. The broad absorption around 3435 cm^−1^ corresponds to O─H stretching from adsorbed water and residual hydroxyl groups on the carbon surface. In the WCS‐SiO_2_ spectrum, the newly emerged bands at 1031 and 780 cm^−1^ correspond to Si─O─Si stretching vibrations, and the band at 1274 cm^−1^ is assigned to Si‐CH_3_ deformation. These characteristic peaks confirm the successful incorporation of the SiO_2_ aerogel.

X‐ray Diffraction (XRD) patterns in Figure 1l for NW and WS exhibit broad characteristic peaks at approximately 15.8° and 22° corresponding to the (101) and (002) crystal planes of cellulose, respectively. These peaks persist in both samples, indicating that the crystalline cellulose structure remains intact after delignification. After carbonization, both WCS and WCS‐SiO_2_ exhibit a broad peak at approximately 24°, which is assigned to the (002) plane of typical amorphous (turbostratic) carbon structures. A weaker peak at around 45°, corresponding to the (100) plane, is also observed, indicating the presence of a small fraction of graphitized carbon. This partial graphitization likely originates from the carbonization of crystalline domains originally present in the cellulose scaffold [22]. Such an amorphous structure endows the material with a lower intrinsic thermal conductivity compared to highly graphitized carbon materials, thereby improving thermal insulation. The attenuation of the weak (100) peak at ∼45° after SiO_2_ incorporation is attributed to the physical dilution and shielding effect of the amorphous SiO_2_ coating, without altering the intrinsic carbon structure [23].

To further investigate the compositional evolution and interfacial interactions, X‐ray photoelectron spectroscopy (XPS) was performed. The survey spectra (Figure S6) reveal that the WS exhibits primary signals for carbon (C 1s at ∼284 eV) and oxygen (O 1s at ∼533 eV). After carbonization, the WCS spectrum shows a markedly intensified C 1s peak and a weakened O 1s signal, reflecting the decomposition of oxygen‐containing functional groups and the formation of a carbon‐rich skeleton. Upon incorporation of the SiO_2_ aerogel, the O 1s intensity increases again, accompanied by the appearance of two new peaks at approximately 102 and 153 eV, corresponding to Si 2p and Si 2s, respectively [24]. Quantitative analysis (Table S1) reveals that the composite contains 39.47 at% C, 37.50 at% O, and 23.03 at% Si. High‐resolution XPS spectra of C 1s and Si 2p were acquired to further elucidate the chemical bonding configurations. The C 1s spectrum of WCS‐SiO_2_ can be deconvoluted into three peaks at approximately 284.8, 286.2 and 287.9 eV, assigned to C─C, C─O, and C═O species, respectively. The Si 2p spectrum of WCS‐SiO_2_ is fitted with two doublets corresponding to Si─C (∼101.8 eV) and Si─O (∼102.9 eV). These emerging Si signals unambiguously confirm the successful incorporation of the SiO_2_ aerogel.

Raman spectroscopy was employed to investigate the structural evolution of the carbon skeleton. As shown in Figure S7a, both NW and WS exhibit a prominent band at approximately 2900 cm^−1^, corresponding to the C‐H stretching vibrations of cellulose and lignin. After carbonization, this band diminishes dramatically into a weak, broad hump, while two intense new peaks emerge at approximately 1350 and 1590 cm^−1^, assigned to the D‐band and G‐band of carbon materials, respectively. The D‐band originates from lattice defects and disordered carbon structures, while the G‐band arises from the in‐plane stretching vibrations of sp^2^‐hybridized graphitic carbon. The intensity ratio of the D‐band to G‐band (I_D_/I_G_) serves as a critical indicator for evaluating the defect density and graphitization degree in carbon‐based materials [25, 26]. The I_D_/I_G_ value of WCS is 0.94, indicating a partially graphitized carbon framework with abundant structural defects inherited from the cellulose precursor (Figure S7b). Upon incorporation of the SiO_2_ aerogel, the I_D_/I_G_ ratio decreases to 0.88. This change is attributed primarily to the interfacial effects introduced by the carbon/SiO_2_ heterogeneous interfaces and the stress imposed by the SiO_2_ coating on the carbon lamellae. These newly introduced interfafces serve as additional polarization centers that contribute positively to the microwave absorption performance.

In summary, the above characterizations confirm that the WCS and WCS‐SiO_2_ composites have been successfully fabricated, faithfully inheriting the intrinsically anisotropic architecture of natural wood. Such pronounced structural anisotropy naturally portends direction‐dependent properties in the resulting materials.

### 2.2 Electromagnetic Wave Absorption Properties

**FIGURE 2:**
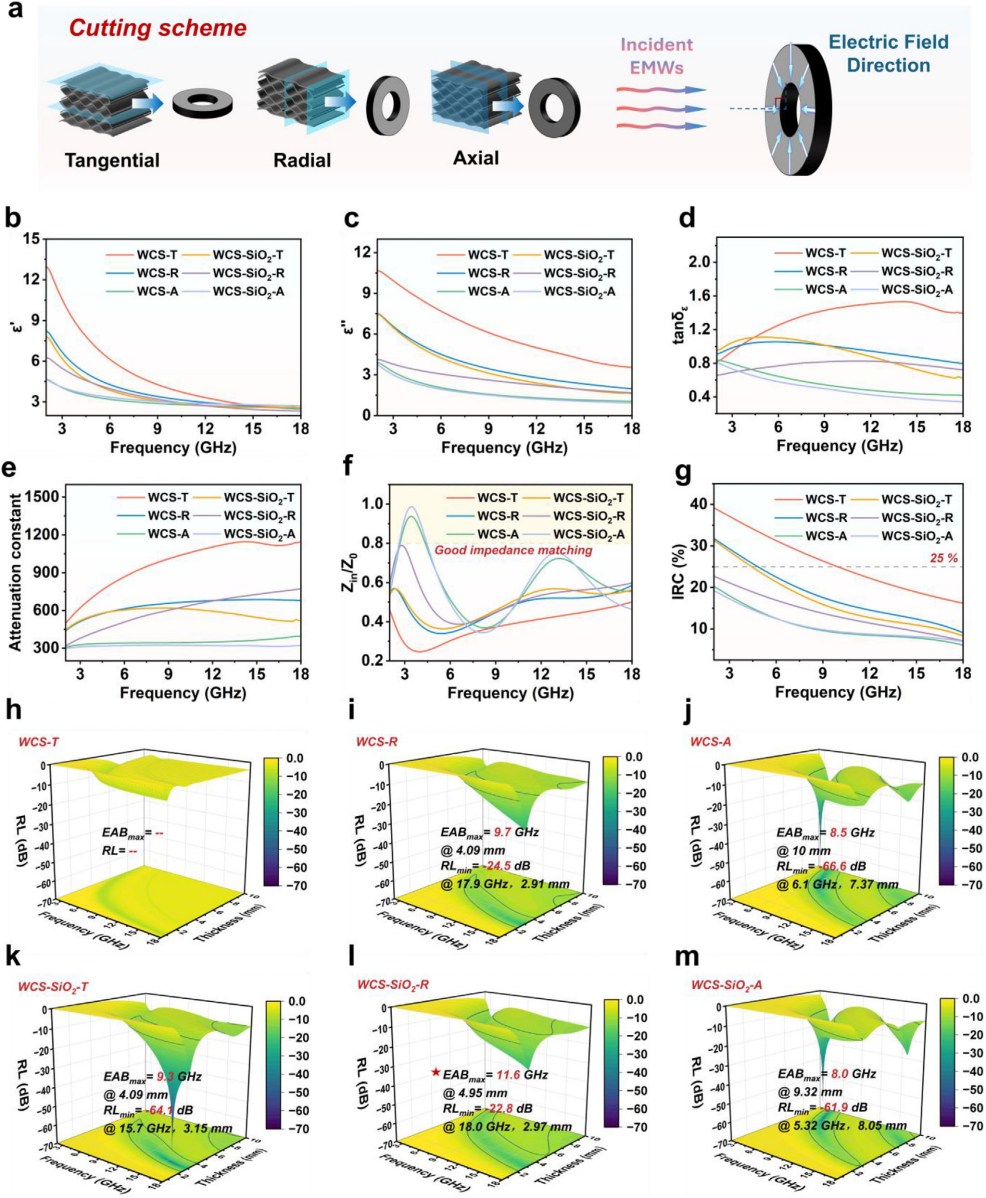
(a) Schematic illustration of the specimen cutting geometry and the alignment of wood anatomical axes with the electric field and propagation direction using the coaxial ring method for anisotropic permittivity determination. The electromagnetic parameters of WCS and WCS‐SiO_2_ in the axial (A), radial (R), and tangential (T) directions over the frequency range of 2–18 GHz. (b) Real part (ε') and (c) imaginary part (ε'') of complex permittivity. (d) Dielectric loss tangent (tan δ). (e) Attenuation constant (α). (f) Impedance matching ratio (Z_in_/Z_0_). (g) Interface reflection coefficient (IRC). Three‐dimensional plots of calculated reflection loss as functions of frequency and thickness for WCS in the (h) tangential, (i) radial, and (j) axial directions, and for WCS‐SiO_2_ in the (k) tangential, (l) radial, and (m) axial directions.

To systematically investigate the electromagnetic wave absorption (EMWA) performance of WCS and WCS‐SiO_2_, their complex permittivity was measured over the frequency range of 2–18 GHz using the coaxial transmission line method. To capture the anisotropic EM response arising from the directionally aligned architecture, all samples were tested along the three principal anatomical directions—axial, radial, and tangential—respectively, with the ring specimens prepared following the protocol schematically illustrated in Figure 2a and Figure S8. Since both WCS and WCS‐SiO_2_ are non‐magnetic, the real part (µ′) and imaginary part (µ′′) of the complex permeability were set to 1 and 0 in the non‐magnetic testing mode. The resulting frequency‐dependent electromagnetic parameters are presented in Figure 2b–g.

As shown in Figure 2b,c, both the real part (ε′) and imaginary part (ε′′) of complex permittivity for all samples exhibit a declining trend with increasing frequency across the 2–18 GHz range, consistent with the typical frequency dispersion behavior observed in carbon‐based absorbers [27, 28]. The real part ε′ represents the material's capacity to store electromagnetic energy, while the imaginary part ε′′ reflects its ability to dissipate or attenuate the incident radiation. Across all samples, the permittivity values follow a consistent directional order: tangential (T) > radial (R) > axial (A). Notably, the substantially higher ε′ and ε′′ values of WCS in the tangential direction imply a highly developed conductive network that, while beneficial for dielectric loss, may risk excessive conductivity and impedance mismatch, potentially impairing microwave absorption. After incorporation of the SiO_2_ aerogel, both ε′ and ε′′ decrease across all three directions, yet the directional ranking (T > R > A) is well preserved. This overall reduction is attributed to the dilution and partial disruption of the conductive carbon network by the insulating SiO_2_ phase, which moderates the permittivity toward a more favorable range for impedance matching.

The dielectric loss tangent (tan δ = ε′′/ε′) represents the ratio of energy dissipated to energy stored per cycle, serving as a direct indicator of the dielectric loss capability of a material [29]. As shown in Figure 2d, in terms of directional dependence, tan δ adheres to the same order of T > R > A, and all values decrease upon incorporation of the SiO_2_ aerogel, consistent with the suppressed conductive network. The attenuation constant α quantifies the attenuation capability of electromagnetic waves per unit propagation distance within the material (Equation S1). As plotted in Figure 2e, α exhibits an overall upward trend with increasing frequency for all samples, signifying enhanced attenuation at higher frequencies. The directional order T > R > A is again preserved. After SiO_2_ aerogel incorporation, α values are moderately reduced across all directions. Notably, α in the tangential direction exhibits a slight downturn above 14 GHz, likely due to the skin effect arising from excessive conductivity, which restricts the effective penetration depth.

Impedance matching, quantified as Z = |Z_in_/Z_0_|, characterizes the ability of electromagnetic waves to enter the material from free space and is a critical prerequisite for subsequent dissipation [30]. Values approaching 1 indicate optimal matching, and the range of 0.8–1.2 is generally regarded as the window of good impedance matching. As shown in Figure 2f, the impedance matching values for both WCS and WCS‐SiO_2_ follow a consistent directional order: T < R < A, with the axial direction exhibiting values closest to 1 and the tangential direction deviating the furthest. After incorporation of the SiO_2_ aerogel, the Z values increase across all three directions, shifting toward the ideal matching range. This improvement demonstrates that introducing SiO_2_ aerogel serves as an effective strategy to modulate the dielectric properties and enhance the impedance matching of carbonized wood‐based absorbers. The interface reflection coefficient (IRC) quantifies the proportion of incident electromagnetic waves reflected at the material surface (Equation S2) [26]. As shown in Figure 2g, the IRC curves of all samples exhibit a monotonically decreasing trend with increasing frequency, similar to the behavior of tan δ and α. The IRC of WCS‐T is notably higher, reaching approximately 39% at 2 GHz, indicative of excessive conductivity‐induced surface reflection that impedes wave entry. In contrast, WCS‐A, WCS‐R, and WCS‐SiO_2_‐A maintain IRC values below 25% across the entire 2–18 GHz range, which is favorable for allowing most incident waves to transmit into the material. For an efficient microwave absorber, it is essential to balance dielectric loss capability with impedance matching while avoiding an excessively high IRC that would prematurely reflect incident radiation.

Reflection loss (RL) and effective absorption bandwidth (EAB), defined as the frequency range where RL ≤ −10 dB (corresponding to >90% microwave energy attenuation), are critical metrics for evaluating microwave absorption performance. Based on transmission line theory and the metal backing model (Equations S3 and S4) [31], the RL values were systematically calculated as functions of frequency and thickness for all samples, and the results are presented as three‐dimensional plots and two‐dimensional contour maps in Figure 2h–m and Figure S9. Among the pure WCS samples, the tangential direction (WCS‐T) exhibits relatively poor absorption owing to its excessively high permittivity, which causes severe impedance mismatch. In contrast, WCS‐R achieves a broad EAB of 9.7 GHz at a thickness of 4.09 mm (Figure 2i; Figure S9b), while WCS‐A delivers a minimum RL of −66.6 dB (at 7.37 mm) with an EAB of 8.5 GHz (at 10 mm) (Figure 2j; Figure S9c). After incorporation of the SiO_2_ aerogel, the absorption performance of the tangential direction is significantly improved: WCS‐SiO_2_‐T attains a minimum RL of −64.1 dB (at 3.15 mm) and a maximum EAB of 9.3 GHz (at 4.09 mm) (Figure 2k; Figure S9d), demonstrating that the introduction of a low‐permittivity SiO_2_ phase effectively optimizes the impedance matching that was previously compromised by excessive dielectric properties. To further evaluate the intrinsic effect of SiO_2_ incorporation, the RL and EAB of WCS and WCS‐SiO_2_ were compared at identical thicknesses across the radial and axial directions (Figure S10). In the radial direction, WCS‐SiO_2_‐R outperforms WCS‐R at thicknesses of 4.09 mm and above, while showing comparable or slightly lower performance at 3 mm. In the axial direction, the EAB of WCS‐SiO_2_‐A is comparable to or slightly lower than that of WCS‐A, which is attributed to the already low intrinsic permittivity of the axial conductive network being further reduced by the insulating SiO_2_ phase. These results demonstrate that SiO_2_ incorporation exerts a direction‐dependent regulatory effect on the microwave absorption performance, consistent with the anisotropic permittivity modulation discussed above. Remarkably, WCS‐SiO_2_‐R realizes an EAB of 11.6 GHz at a thickness of only 4.95 mm (Figure 2l; Figure S9e), fully covering the X and Ku bands and outperforming most reported carbon aerogels. This high performance is attributed to the optimized material design, which simultaneously enhances electromagnetic loss capability and achieves better impedance matching across the measured frequency range.

To further rationalize the relationship between the optimal thickness and the matching frequency, the quarter‐wavelength (λ/4) interference cancellation model was employed. The λ/4 theoretical thickness curve was calculated and superimposed onto the RL contour map of WCS‐SiO_2_‐R (Figure S11). As shown in the figure, the absorption peaks at different thicknesses systematically redshift with increasing thickness, consistent with the inverse relationship between the operating frequency and the wavelength. Notably, the absorption maxima at various thicknesses maintain precise frequency alignment with their respective optimal impedance matching points, and the vertical connections between these experimental peaks and their corresponding thickness markers intersect precisely with the λ/4 contour, providing strong experimental validation of the interference cancellation mechanism. Secondary absorption peaks are also observed at theoretical thicknesses corresponding to the three‐quarter wavelength (3λ/4) condition. These results confirm that the enhanced absorption of WCS‐SiO_2_‐R is governed by the λ/4 interference cancellation mechanism under favorable impedance matching conditions.

**FIGURE 3:**
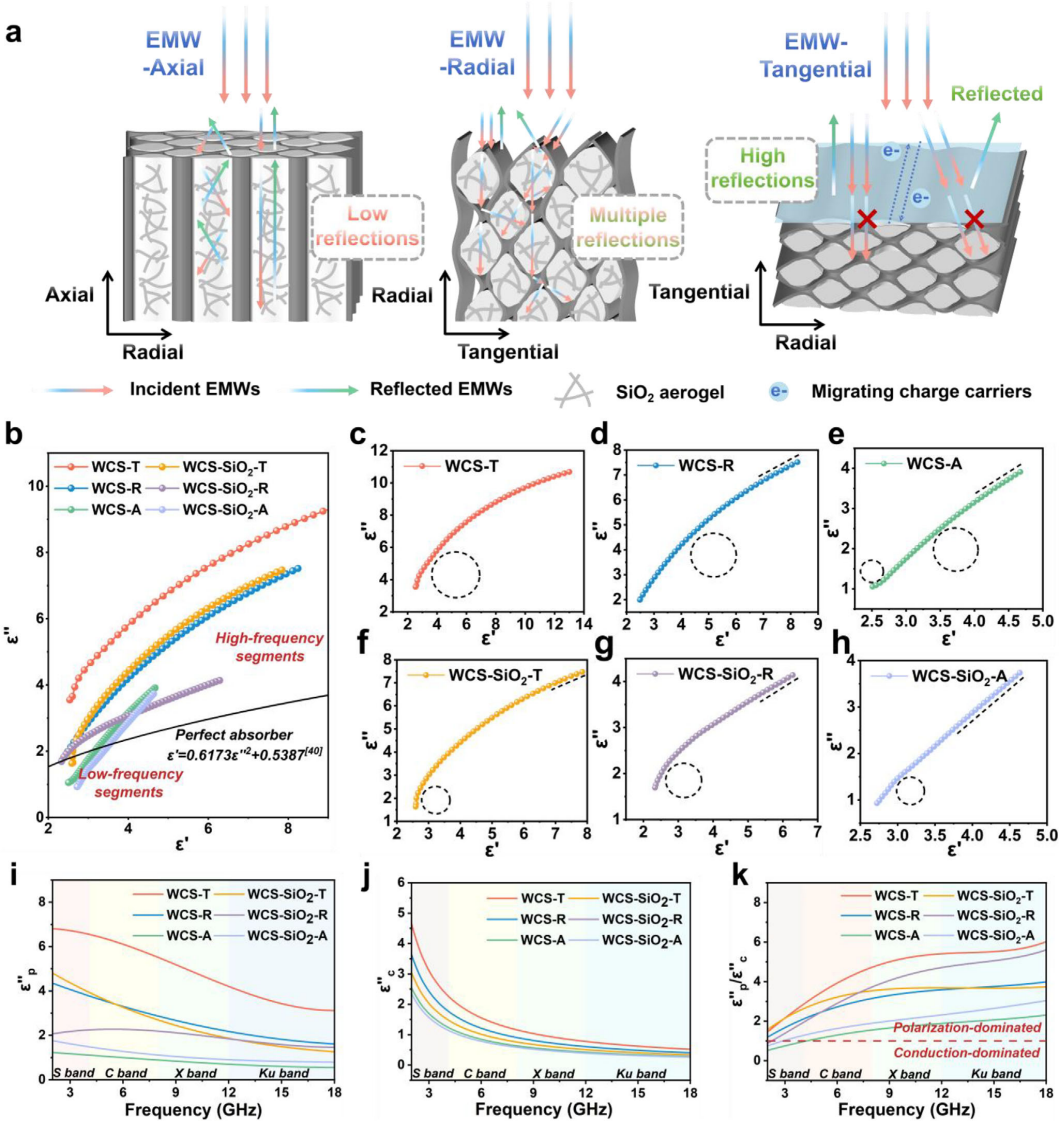
(a) The schematic illustration of the anisotropic microwave absorption mechanisms of WCS‐SiO_2_ along the tangential, radial, and axial directions. (b) Cole–Cole plots of all samples and the perfect absorption curve for dielectric microwave absorbers. (c–h) Enlarged Cole–Cole plots of each individual sample. (i–k) Frequency‐dependent conductive loss, polarization loss, and the ratio of polarization loss to conduction loss for different samples.

To elucidate the origin of the direction‐dependent microwave absorption performance, the anisotropic interaction between the incident electromagnetic waves and the WCS architecture is schematically illustrated in Figure 3a. In the axial direction, the electromagnetic wave propagates directly into the open, interconnected channels between the stacked lamellar layers. According to the right‐hand rule [32], the electric field vector oscillates perpendicular to the lamellar layers, and the induced charge transport is accordingly oriented normal to the layer planes. Consequently, charge transport is repeatedly interrupted by the interlayer interfaces, resulting in a poorly connected conductive network and intrinsically low conductivity. Owing to the open pathway and limited obstructive interfaces along this direction, the wave encounters few reflecting sites, yielding a low interface reflection coefficient and favorable impedance matching. However, the dielectric loss capability is insufficient due to the discontinuous conduction paths, leading to only moderate overall absorption despite the ease of wave entry. In the tangential direction, the electromagnetic wave impinges perpendicularly onto the large planar surfaces of the lamellae layers. The electric field component drives charge carriers to move unimpeded within the continuous conductive lamellar plane, causing massive charge accumulation at the lamellar surfaces and giving rise to excessively strong interfacial polarization and an overdeveloped conductive network. The resulting impedance mismatch is so severe that most incident radiation is reflected at the surface before penetration. Hence, the tangential direction exhibits the highest permittivity values but the poorest absorption performance. In the radial direction, although the incident wave also propagates parallel to the lamellar layers, the undulating and curved morphology of the arch‐shaped lamellae forces the wave to undergo multiple internal reflections and scattering once it enters the material. This structural tortuosity effectively prolongs the propagation path and creates abundant polarization sites without establishing a continuous conductive network as strong as that in the tangential direction. As a result, the electromagnetic parameters in the radial direction fall into an optimal balance window, where both dielectric loss capability and impedance matching are satisfied simultaneously, yielding the largest effective absorption bandwidth among the three directions. After incorporation of the SiO_2_ aerogel, the absorption performance, particularly in the radial direction, is further enhanced. The SiO_2_ nanoparticles uniformly coat the carbon lamellae and partially fill the interlayer voids, introducing additional carbon/SiO_2_ heterogeneous interfaces. These newly created interfaces serve as extra polarization centers, intensifying interfacial polarization relaxation and further boosting the dielectric loss capacity. Meanwhile, the insulating SiO_2_ phase moderately suppresses the excessive conductivity, optimizing the impedance matching. This synergistic effect enables WCS‐SiO_2_‐R to achieve an effective absorption bandwidth of 11.6 GHz at a thickness of only 4.95 mm, fully covering the X and Ku bands.

To further elucidate the underlying electromagnetic loss mechanisms, the dielectric relaxation behavior of the samples was analyzed based on the Debye relaxation theory (Equation S5) [33]. In a Cole–Cole plot, the real part (ε′) and imaginary part (ε′′) of the complex permittivity are plotted on the x‐ and y‐axes, respectively. Each Debye‐type relaxation process manifests as a semicircle [34], whereas conduction loss appears as a linear tail at higher ε′ values [35, 36, 37]. By examining the relative prominence of semicircles and linear segments, the dominant loss mechanism—whether polarization relaxation or conduction loss—can be qualitatively assessed. Figure 3b presents the Cole–Cole plots for all samples, together with the perfect absorption curve for dielectric microwave‐absorbing materials [38], which defines the ideal ε′–ε′′ relationship for achieving strong absorption. The intersection or proximity of the Cole–Cole curves to this ideal line indicates that the material possesses favorable dielectric properties conducive to strong microwave absorption.

Cole–Cole plots of all samples are presented in Figure 3b, together with the perfect absorption curve for dielectric microwave absorbers. As shown in Figure 3b, the data points of all samples except WCS‐T intersect with the perfect absorption curve [39]. Notably, WCS‐R, WCS‐SiO_2_‐T, and WCS‐SiO_2_‐R intersect the curve in the low‐ε′ region, suggesting that their strongest absorption peaks occur at high frequencies, consistent with the RL results discussed previously. Figure 3c–e presents the enlarged Cole–Cole plots of the pure WCS samples. The WCS‐T curve appears as a single arc with pronounced curvature, indicative of a dominant polarization loss mechanism arising from strong interfacial charge accumulation at the conductive lamellar surfaces. In contrast, WCS‐R and WCS‐A display a nearly linear relationship in the low‐frequency region, demonstrating that conduction loss, driven by electron migration within the carbon conductive network, dominates at lower frequencies. At higher frequencies, both curves deviate from linearity and develop discernible curvature, signaling the increasing contribution from polarization relaxation processes. This transition from conduction‐dominated to polarization‐enhanced loss with increasing frequency is characteristic of carbon‐based absorbers with hierarchical architectures. Figure 3f–h displays the Cole–Cole plots of the WCS‐SiO_2_ samples. Compared with their pure WCS counterparts, all three WCS‐SiO_2_ samples exhibit noticeably enhanced curvature in the high‐frequency region, indicating an increased contribution from polarization loss following SiO_2_ aerogel incorporation. This enhancement is attributed to the introduction of abundant carbon/SiO_2_ heterogeneous interfaces, which serve as additional polarization centers for interfacial polarization relaxation, and to the increased defect sites associated with the SiO_2_ coating that promote dipole polarization. The more pronounced deviation from linearity in WCS‐SiO_2_ thus confirms the synergistic effect of conduction loss and polarization loss enabled by the dual‐network architecture.

To quantitatively assess the relative contributions of conduction loss and polarization loss to the overall dielectric loss, the imaginary part of the complex permittivity (ε′′) was separated into conduction loss (ε′′_c_) and polarization loss (ε′′_p_) components based on the Debye theory [40]. Nonlinear least‑squares fitting was performed on the ε′′ spectra (Equation S6), and the resulting ε′′_p_ and ε′′_c_ values are presented in Figure 3i,j, respectively. The frequency‑dependent ratio ε′′_p_/ε′′_c_, which serves as a quantitative indicator of the dominant loss mechanism, is plotted in Figure 3k. A ratio greater than 1 indicates polarization‑dominated loss, while a ratio below 1 signifies conduction‑dominated loss. For pure WCS, the tangential direction (WCS‑T) exhibits ε′′_p_ values substantially higher than ε′′_c_ across the entire frequency range (Figure 3k). This confirms that polarization loss dominates in the tangential direction, consistent with the strong interfacial charge accumulation on the highly conductive lamellar surfaces, as discussed in the Cole–Cole analysis. The radial direction (WCS‑R) also shows polarization‑dominated loss, with ε′′_p_/ε′′_c_ remaining above 1 throughout the measured frequency range. Such a pronounced polarization contribution in the radial direction arises from the undulating lamellar architecture, which forces the incident wave to undergo multiple internal reflections and creates abundant carbon–air interfaces that promote interfacial polarization relaxation. In contrast, the axial direction (WCS‐A) exhibits a distinct frequency‐dependent behavior: the ε′′_p_/ε′′_c_ ratio remains below 1 in the low‐frequency S‐band, then rises above 1 from the C‐band onward and continues to increase with frequency. This trend indicates that polarization loss ultimately dominates across most of the measured range, yet the overall contribution of conduction loss is noticeably higher than in the radial and tangential directions, consistent with the relatively continuous electron transport pathways along the axial channels. After incorporation of the SiO_2_ aerogel, the polarization‐to‐conduction loss ratio evolves differently across the three directions. For the tangential direction, the ε′′_p_/ε′′_c_ ratio decreases upon SiO_2_ incorporation. This can be understood as follows: in pristine WCS‐T, the incident wave is largely reflected at the surface due to severe impedance mismatch caused by the highly conductive lamellar plane, and the measured dielectric response is dominated by intense interfacial charge accumulation at the surface. The introduction of the low‐permittivity SiO_2_ aerogel alleviates this impedance mismatch, allowing greater wave penetration into the interior. Consequently, the surface‐confined polarization is partially relieved, and the interior conductive network is activated, leading to a relative decline in the polarization‐to‐conduction loss ratio. In contrast, both the radial and axial directions exhibit an increased ε′′_p_/ε′′_c_ ratio after SiO_2_ incorporation. This enhancement is attributed to the newly introduced carbon/SiO_2_ heterogeneous interfaces, which serve as additional polarization centers for interfacial polarization relaxation, as well as to the defect sites associated with the SiO_2_ coating that promote dipole polarization. These quantitative trends are fully consistent with the qualitative features revealed by the Cole–Cole plots, confirming that the SiO_2_ aerogel plays distinct yet complementary roles along different directions in modulating the loss mechanisms.

**FIGURE 4:**
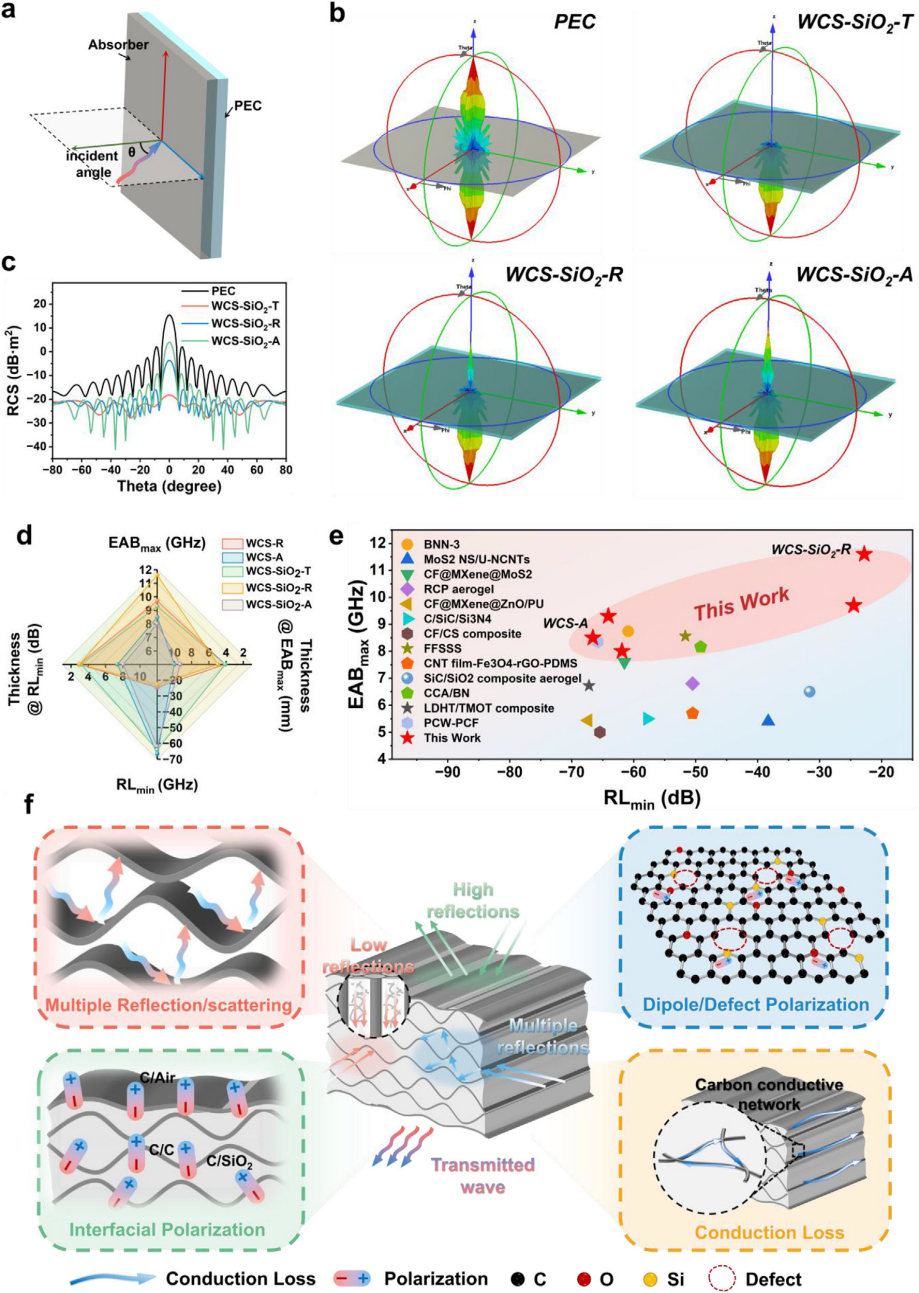
(a) The schematic of the square model used for RCS simulations. (b) The 3D bistatic RCS patterns of PEC, WCS‐SiO2‐T, WCS‐SiO2‐R, and WCS‐SiO2‐A. (c) Monostatic RCS values compared. (d) The summary chart of the microwave absorption performance of various samples. (e) The comparison of the microwave absorption performance of single‐layer absorbers reported recently in terms of RLmin and EAB. (f) The schematic of the EMWA mechanism of WCS‐SiO_2_.

To further evaluate the practical stealth capability of the WCS‐SiO_2_ composites, radar cross‐section (RCS) simulations were performed with the time‐domain solver under far‐field and open boundary conditions. The simulation model is a square perfect electric conductor (PEC) substrate coated with the WCS‐SiO_2_ absorber layer (Figure 4a). The corresponding 3D bistatic RCS patterns of the bare PEC and the three WCS‐SiO_2_ samples are presented in Figure 4b. The bare PEC substrate exhibits pronounced electromagnetic reflection, whereas all WCS‐SiO_2_ samples demonstrate effective wave attenuation. Figure 4c compares the monostatic RCS values of the three orientations with that of the bare PEC. In general, a smaller RCS value represents more efficient far‐field electromagnetic wave attenuation. Notably, WCS‐SiO_2_‐T achieves the best RCS reduction among the three orientations, with its monostatic RCS values remaining below −10 dB·m^2^ across the entire angular range from −80° to 80°, indicating promising practical microwave absorption performance under the unified simulation conditions.

Subsequently, separate simulations were performed using the individually optimized thickness and frequency for each orientation to further demonstrate the maximum stealth potential achievable along each direction: 3.15 mm at 15.7 GHz for the tangential direction, 2.97 mm at 18 GHz for the radial direction and 8.05 mm at 5.35 GHz for the axial direction. The RCS results for the radial and axial directions are provided in Figures S12 and S13. Compared with the strongly reflective PEC plate, all WCS‐SiO_2_ samples show markedly reduced RCS values over a wide angular range, confirming effective radar wave attenuation. Under these application‐specific optimal conditions, the tangential direction achieves the most pronounced RCS reduction, demonstrating its high far‐field microwave absorption capability when the thickness and frequency are individually optimized.

Figure 4d,e and Table S3 compare the EMWA performances of WCS‐SiO_2_ composite with recently reported microwave absorbers [41,42, 43, 44, 45, 46, 47, 48, 49, 50, 51, 52, 53]. In particular, WCS‐SiO_2_‐R achieves an EAB of 11.6 GHz at a thickness of 4.95 mm, placing it among the most competitive candidates. Figure 4f illustrates the microwave absorption mechanism of WCS‐SiO_2_ composite, highlighting multiple synergistic loss pathways ranging from macroscopic structural effects to microscopic dielectric responses:

1. At the atomic‐to‐nanoscale, dipole/defect polarization and conduction loss dominate. The residual oxygen‐containing functional groups and structural defects in the partially graphitized carbon skeleton, together with the silanol (Si─OH) groups and oxygen vacancies associated with the SiO_2_ aerogel, act as permanent dipoles. Under the high‐frequency electromagnetic field, these dipoles undergo repeated orientation and relaxation, converting electromagnetic energy into thermal energy. Simultaneously, the interconnected carbon conductive network enables directional charge carrier migration, forming oscillating currents that dissipate energy through Joule heating. The moderate conductivity achieved at 680°C carbonization prevents excessive reflectivity while maintaining sufficient dielectric loss [54, 55, 56].
2. At the nanoscale, interfacial polarization arises from abundant heterogeneous interfaces—including C/C, C/air, and C/SiO_2_ interfaces—where space charges accumulate under the alternating electric field. The newly introduced C/SiO_2_ interfaces are particularly effective in intensifying interfacial polarization relaxation, contributing significantly to the enhanced dielectric loss [57, 58, 59].
3. At the microscale, the porous, lamellar architecture inherited from the wood sponge facilitates extensive multiple reflections and scattering of incident electromagnetic waves within the interlayer voids and undulating channels. This structural tortuosity prolongs the propagation path and traps electromagnetic energy, increasing the probability of subsequent dissipation by the aforementioned nanoscale mechanisms. These multi‐scale loss pathways are further governed by the intrinsic structural anisotropy of the wood‐derived carbon skeleton, which dictates distinct electromagnetic responses along the three principal directions. The SiO_2_ aerogel modulates this anisotropy by suppressing excessive surface reflectivity in the tangential direction while enhancing polarization loss in the radial and axial directions, effectively narrowing the performance gap among the three orientations [60, 61, 62].

Collectively, a self‐reinforcing dissipation network is established through this hierarchical synergy, spanning from atomic‐scale dipoles and nanoscale interfaces to microscale structural scattering, thereby maximizing electromagnetic energy conversion. The integration of intrinsic structural anisotropy with SiO_2_ modulation further enables balanced broadband and strong absorption across all directions, thereby realizing high‐performance microwave absorption in the WCS‐SiO_2_ composite.

### 2.3 Mechanical and Thermal Insulation Properties

**FIGURE 5:**
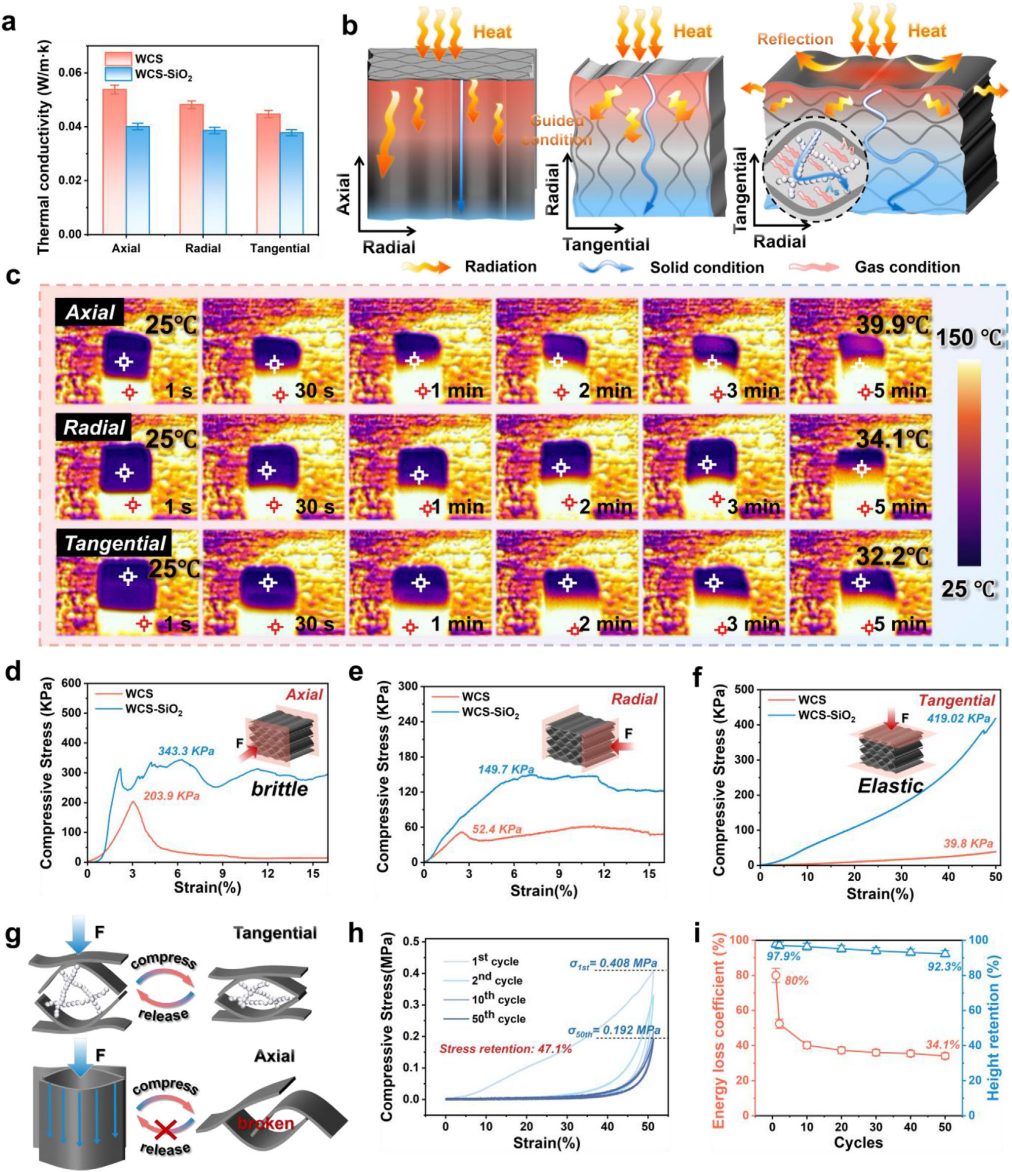
(a) The thermal conductivities of WCS and WCS‐SiO_2_. (b) Schematic illustration of the anisotropic heat transfer mechanisms of WCS‐SiO_2_. (c) The comparison of axial, radial, and tangential infrared images on hot plate of 150°C for 5 min. (d–f) The compressive stress–strain curves of WCS and WCS‐SiO_2_ in the axial, radial, and tangential directions. (g) The schematic illustration of anisotropic mechanical properties. (h) The stress–strain fatigue curves for the 50 loading‐unloading cycles of WCS‐SiO_2_. (i) Energy loss coefficient and height retention of WCS‐SiO_2_ in the test.

The thermal insulation properties of WCS and WCS‐SiO_2_ were evaluated by measuring the thermal conductivity along the three principal anatomical directions. As shown in Figure 5a, the WCS exhibits pronounced anisotropic thermal transport behavior, with thermal conductivities of 0.054, 0.048, and 0.044 W/(m·K) in the axial, radial, and tangential directions, respectively. After incorporation of the SiO_2_ aerogel, the thermal conductivity decreases further across all three directions to 0.040, 0.038, and 0.037 W/(m·K), respectively, while the directional difference is notably diminished. To further visualize the anisotropic thermal insulation performance, the WCS‐SiO_2_ composite was placed on a 150°C heating stage for 5 min, and the surface temperature evolution was recorded along the three principal directions (Figure 5c; Figure S14). Consistent with the thermal conductivity measurements, the tangential direction exhibits the best thermal insulation, with the top surface temperature rising from 25°C to only 32.2°C after 5 min. The radial direction shows intermediate performance, reaching 34.1°C, while the axial direction displays the weakest insulation, attaining 39.9°C. This directional trend mirrors the thermal conductivity results and further confirms the anisotropic heat transfer behavior inherited from the wood‐derived architecture.

Figure 5b illustrates the anisotropic thermal insulation mechanism of WCS and its further enhancement after SiO_2_ incorporation. In the axial direction, the carbonized wood fibers retain their preferential alignment inherited from the tree growth direction, forming relatively continuous solid conduction pathways that guide heat transport efficiently along the oriented fiber axis, contributing to the highest thermal conductivity among the three directions. In contrast, the tangential direction offers superior thermal resistance. The lamellar layers are oriented perpendicular to the incident heat flux, acting as multiple reflective barriers that suppress thermal radiation and restrict gas convection. Heat absorbed at the surface is preferentially redirected along the axial channels, dissipating laterally away from the heat source. Consequently, the tangential direction exhibits the lowest thermal conductivity and the best thermal insulation performance. The radial direction displays intermediate thermal transport behavior. Although the lamellar layers are oriented parallel to the heat flux in this direction, the undulating and partially discontinuous nature of these layers introduces interfacial thermal resistance, partially impeding both solid and gaseous conduction. After incorporation of the SiO_2_, the thermal conductivity decreases further across all directions and the anisotropy is notably diminished. This can be attributed to the suppression of both gaseous and solid‐phase heat transfer. For the gaseous phase, the nanoporous network introduced by the SiO_2_ aerogel creates numerous pores smaller than the mean free path of gas molecules, triggering the Knudsen effect [63, 64]. Under this condition, gas molecules collide more frequently with pore walls than with each other, significantly reducing gaseous conduction. For the solid phase, the SiO_2_ nanoparticles form a discontinuous network that forces heat to travel along extended, tortuous pathways. Combined with the inherently low crystallinity of the amorphous carbon and silica phases, solid conduction is effectively suppressed. Furthermore, the uniform filling of SiO_2_ within the lamellar voids and channels homogenizes the composite architecture, thereby weakening the structural anisotropy and reducing the directional variation in thermal conductivity.

Robust mechanical performance is essential for the practical application of thermal insulation materials. The mechanical properties of WCS and WCS‐SiO_2_ were evaluated by compressive tests along the three directions. Figure 5d–f presents the corresponding compressive stress–strain curves, and Figure S15 displays optical images of the specimens before, during, and after compression. For WCS, the axial direction exhibits a peak stress of 203.9 kPa at a strain of only ∼3%, followed by a rapid drop, characteristic of a brittle failure mode. In contrast, the tangential direction displays a typical elastic response, sustaining a stress of 39.8 kPa at 50% strain while maintaining structural integrity without fracture. The radial direction shows intermediate behavior: after reaching 52.4 kPa at approximately 2.7% strain, the stress stabilizes into a plateau with minor oscillations, reflecting a balance between structural compliance and resistance. After incorporation of the SiO_2_ aerogel, the compressive stress–strain profiles of WCS‐SiO_2_ retain the directional trends observed in WCS, while the overall stress levels increase across all directions. Furthermore, the tangential direction undergoes a pronounced enhancement, reaching 419 kPa at 50% strain—an order of magnitude higher than that of WCS. Figure 5g illustrates the anisotropic deformation mechanisms of WCS and WCS‐SiO_2_ under compression along the tangential and axial directions. In the tangential direction, the compressive load is applied perpendicular to the lamellar layers, causing the arch‐shaped carbon skeleton to undergo bending, buckling, and interlayer sliding without structural rupture. The undulating lamellae effectively redistribute stress through large‐strain flexural deformation, enabling the material to sustain high compressive strain while maintaining structural integrity. Upon unloading, the elastic energy stored in the deformed lamellae drives recovery, imparting the tangential direction with high compressibility and resilience. In contrast, axial compression applies load parallel to the oriented carbon fibers and stacked lamellar edges. The stress concentrates along the relatively rigid fiber axis and interlayer interfaces, where the limited capacity for bending deformation leads to abrupt structural collapse and crack propagation, resulting in the characteristic brittle failure at low strain. Upon incorporation of the SiO_2_ aerogel, the mechanical performance is enhanced across all directions while the fundamental deformation characteristics are preserved. The SiO_2_ nanoparticles, uniformly coating the carbon lamellae and filling the interlayer voids, act as a reinforcing phase that strengthens the lamellar walls and provides additional load‐bearing support. In the tangential direction, the SiO_2_ network interconnects adjacent lamellae, effectively suppressing excessive interlayer sliding and pore collapse, which accounts for the pronounced tenfold enhancement in compressive stress at 50% strain. In the axial and radial directions, the SiO_2_ coating reinforces the carbon framework and partially mitigates stress concentration, leading to a moderate increase in strength. The dual‐network architecture thus synergistically combines the structural compliance of the lamellar carbon skeleton with the reinforcing function of the SiO_2_ aerogel, transforming the direction‐dependent mechanical response into a tunable property.

To further evaluate the long‐term mechanical stability along the most compliant direction, cyclic compression tests were performed on WCS‐SiO_2_ in the tangential direction at 50% strain for 50 cycles. After 50 cycles, the sample retains a height retention of 92.3% and an energy loss coefficient of approximately 34.1%, with a stress retention of 47.1%. The residual strain and cycle‐to‐cycle energy dissipation stabilize at approximately 24% and 0.375 MJ/m^3^, respectively (Figure 5h,i; Figure S16). These results demonstrate favorable compressibility and stable cyclic response under repeated large‐strain deformation.

## 3 Conclusions

In summary, we fabricated a SiO_2_‐aerogel‐incorporated wood carbon sponge that inherits the anisotropic architecture of natural wood. A robust permittivity ordering (tangential > radial > axial) was revealed. With the incorporation of SiO_2_ aerogel, the composite achieves markedly enhanced overall performance, including an EAB of 11.6 GHz at 4.95 mm thickness in the radial direction (among the highest reported for carbon aerogels), and a RLmin of −64.1 dB at 3.15 mm in the tangential direction. It also exhibits low and direction‐dependent thermal conductivity (0.037‐0.040 W/(m·K)) and pronounced anisotropic compressibility—the tangential direction sustains 50% strain at 0.42 MPa, whereas the axial direction fails at only 6.5% strain with a stress of 0.34 MPa. In terms of overall application potential, the tangential direction appears most promising. It delivers the best thermal insulation and mechanical compressibility, and its microwave absorption is substantially improved to a competitive level after SiO_2_ incorporation. This work reveals that the intrinsic structural anisotropy of natural wood can be utilized as a design parameter to simultaneously tailor electromagnetic, thermal, and mechanical responses. By selecting the most suitable working direction according to specific requirements, the material enables application‐oriented customization. This offers a promising route toward multifunctional lightweight composites for stealth and thermal insulation applications.

## 4 Experimental Section

### 4.1 Materials

Balsa wood was purchased from Shaoguan, Guangdong Province. Sodium chlorite (NaClO_2_, 80%), Cetyl trimethyl ammonium bromide (CTAB), Methyltrimethoxysilane (MTMS, 98%), Ammonium hydroxide solution (NH_3_·H_2_O, 25–28%) were bought from Macklin Co., Ltd. Ethanol (95%) and acetic acid (≥99.5%) were purchased from Sinopharm Chemical Reagent Co., Ltd. All the chemical reagents were used as received. The deionized water was lab‐made.

### 4.2 Preparation of WS

The delignification process was conducted using an acidified sodium chlorite method. Typically, balsa wood blocks (20 mm × 20 mm × 20 mm) were immersed in a 2 wt.% sodium chlorite aqueous solution, adjusted to pH 4.5 with acetic acid, and treated in a water bath at 80°C for 8 h. This procedure was repeated twice with fresh solution. After delignification, the samples were thoroughly washed with deionized water at 80°C for 5 h to remove residual chemicals. The resulting wood sponges were then frozen at −40°C for 4 h, followed by freeze‐drying for 48 h.

### 4.3 Preparation of WCS‐SiO_2_

The WS was carbonized at 680°C for 1 h under a flowing argon atmosphere to obtain the WCS. The SiO_2_ sol was prepared by dissolving CTAB in deionized water, followed by the addition of MTMS and continuous stirring to obtain a homogeneous sol. The WCS was then immersed in the sol and subjected to vacuum impregnation for 15 min. Subsequently, NH_3_·H_2_O was introduced to catalyze the gelation. After aging for 1 day, the resulting composite hydrogel was subjected to solvent exchange with anhydrous ethanol, followed by ambient pressure drying at 90°C for 2 days to yield the WCS‐SiO_2_ aerogel composite.

### 4.4 Characterization

The bulk density (ρ) was calculated from the weight and physical dimensions of the sample. The linear shrinkage was calculated by comparing the dimensions of the sample before and after drying or carbonization. The morphologies were analyzed by scanning electron microscopy (SEM, Hitachi S‐3800 and ZEISS Sigma 300). The chemical structures were investigated by Fourier transform infrared (FTIR, Vertex 70), X‐ray photoelectron spectroscopy (XPS, Thermo Scientific K‐Alpha), and Raman spectroscopy (Horiba LabRAM HR Evolution), and X‐ray diffraction (XRD; Rigaku SmartLab SE, Japan). The compressive mechanical properties were measured using an electronic universal testing machine (FL4204GL, Fule Test Technology) with a displacement rate of 0.5 mm·min^−1^. The room‐temperature thermal conductivities were measured by the Hot‐Disk method (Hot Disk TPS 3500).Specimens (20 × 20 × 10 mm^3^) were measured in the isotropic mode (10 mW, 20 s) with the 10 mm dimension aligned along the probing direction. Five independent measurements were performed for each direction (RSD < 3%). For electromagnetic characterization, aerogels were infiltrated with paraffin wax under vacuum and then precision‐cut into coaxial rings (outer diameter: 7.00 mm; inner diameter: 3.04 mm). Complex permittivity and permeability were measured over 2–18 GHz using a vector network analyzer (ZNA43, Rohde & Schwarz, Germany).

## Author Contributions

**Shuo Sun**: conceptualization, investigation, funding acquisition, Writing – original draft, visualization, validation, methodology, project administration, formal analysis, software. **Zhimeng Zhao**: conceptualization, investigation, formal analysis, project administration, methodology. **Weikai Zhan**: conceptualization, investigation, validation, methodology, software, formal analysis. **Yonggang Jiang**: investigation, funding acquisition, conceptualization, visualization, validation, project administration, resources, supervision. **Fengqi Liu**: conceptualization, investigation, validation, methodology, project administration, resources, data curation, formal analysis. **Yijie Hu**: writing – review and editing, conceptualization, investigation, funding acquisition, visualization, validation, methodology, project administration, formal analysis, resources, supervision, data curation. **Junzong Feng**: funding acquisition, investigation, conceptualization, writing – review and editing, visualization, validation, project administration, resources, supervision, formal analysis. **Jian Feng**: conceptualization, investigation, funding acquisition, visualization, methodology, validation, formal analysis, software, resources, data curation.

## Conflicts of Interest

The authors declare no conflicts of interest.

## Supporting information




**Supporting File**: advs77888‐sup‐0001‐SuppMat.docx.

## Data Availability

The data that supports the findings of this study are available in the supplementary material of this article.

## References

[1] J. Lin , L. Wang , W. Chu , et al., “Ultrabroadband Low‐Frequency Microwave Absorption in Multiscale Aerogel‐Metamaterial Hybrids,” Advanced Functional Materials 36, no. 36 (2026): 27401, 10.1002/adfm.202527401.

[2] M. He , X. Bi , S. Liang , et al., “Mechanically Programmable Microwave Absorption in a Dual‐Gradient Cellulose‐Based Aerogel,” Advanced Functional Materials 36, no. 49 (2026): 75806, 10.1002/adfm.75806.

[3] S. Li , Y. Du , H. Ye , et al., “All‐Natural Self‐Bonded Biocomposite Providing Superior Electromagnetic Interference Shield Performance With Effective Absorption,” Advanced Functional Materials 34, no. 41 (2024): 2406282, 10.1002/adfm.202406282.

[4] Z. Lou , Q. Wang , U. I. Kara , et al., “Biomass‐Derived Carbon Heterostructures Enable Environmentally Adaptive Wideband Electromagnetic Wave Absorbers,” Nano‐Micro Letters 14 (2022): 11. 10.1007/s40820-021-00750-z PMC8643388 34862949

[5] J. Wei , X. Sun , Q. Gao , et al., “Hollow CoFe‐MOF@Bamboo Derived Lightweight Carbon Composites for High‐Efficiency Electromagnetic Interference Shielding and Thermal Management,” Advanced Functional Materials 36, no. 7 (2026): 13845, 10.1002/adfm.202513845.

[6] C. Wang , Z. Zhong , C. Hu , et al., “Flash Joule Heating Enabling MoS_2_/Carbon Composites from Waste Cotton Fabric with Tunable Interfaces for Electromagnetic Wave Absorption,” Journal of Materials Science & Technology 277 (2026): 319–330.

[7] Y. Cheng , J. Z. Y. Seow , H. Zhao , Z. J. Xu , and G. Ji , “A Flexible and Lightweight Biomass‐Reinforced Microwave Absorber,” Nano‐Micro Letters 12 (2020): 125. 10.1007/s40820-020-00461-x PMC7770825 34138152

[8] J. Lee , J. Choi , J. Seo , et al., “Reinforced Concrete‐Inspired Multiscale Hierarchical Metamaterial Composite for Synergistic Enhancement Across Thermal, Electromagnetic, and Mechanical Domains,” Advanced Functional Materials 36, no. 5 (2026): 20521, 10.1002/adfm.202520521.

[9] S. Park , J. Choi , J. Lee , et al., “Gradient Porous and Carbon Black‐Integrated Cellulose Acetate Aerogel for Scalable Radiative Cooling,” Small 21, no. 7 (2025): 2409873, 10.1002/smll.202409873. 39777858

[10] A. H. Asif , L. Shi , T. Ding , et al., “Emerging Engineered Biochar for Environmental and Energy Applications,” The Journal of Bioresources and Bioproducts 10, no. 1 (2025): 1–3.

[11] G. Shao , R. Xu , Y. Chen , et al., “Miniaturized Hard Carbon Nanofiber Aerogels: From Multiscale Electromagnetic Response Manipulation to Integrated Multifunctional Absorbers,” Advanced Functional Materials 34, no. 48 (2024): 2408252, 10.1002/adfm.202408252.

[12] G. Shao , Y. Yang , S. Jia , et al., “Covalent Organic Framework‐Amplified Polarization Loss in Ultralight Schottky Heterojunction Aerogels for Low‐Frequency Electromagnetic Wave Absorption,” Advanced Functional Materials 36, no. 10 (2026): 16078, 10.1002/adfm.202516078.

[13] T. Zhang , B. Bai , G. Shao , Y. Du , R. Fu , and H. Sai , “Hierarchically Porous Aerogels With Heterogeneous Nanodomains Constructed From Sub‐1 Nm Nanowires for Efficient Microwave Absorption,” Nano Research 19 (2026): 94908689.

[14] R. Xu , J. Chen , N. Yan , et al., “High‐Value Utilization of Agricultural Residues Based on Component Characteristics: Potentiality and Challenges,” Journal of Bioresources and Bioproducts 10 (2025): 271–294.

[15] C. Chen , J. Song , S. Zhu , et al., “Scalable and Sustainable Approach Toward Highly Compressible, Anisotropic, Lamellar Carbon Sponge,” Chem 4 (2018): 544.

[16] J. Song , C. Chen , S. Zhu , et al., “Processing Bulk Natural Wood Into a High‐Performance Structural Material,” Nature 554, no. 7691 (2018): 224–228, 10.1038/nature25476. 29420466

[17] C. Chen , Y. Kuang , S. Zhu , et al., “Structure–Property–Function Relationships of Natural and Engineered Wood,” Nature Reviews Materials 5, no. 9 (2020): 642–666, 10.1038/s41578-020-0195-z.

[18] Z. Wu , H.‐W. Cheng , C. Jin , et al., “Dimensional Design and Core–Shell Engineering of Nanomaterials for Electromagnetic Wave Absorption,” Advanced Materials 34, no. 11 (2022): 2107538, 10.1002/adma.202107538. 34755916

[19] T. Ji , H. Sun , B. Cui , et al., “Lightweight and Superelastic Wood Carbon Sponges Enabled by Wood Cell Wall Reconfiguration,” Advanced Materials 37, no. 34 (2025): 2504980, 10.1002/adma.202504980. 40484920

[20] X. Zhao , Y. Liu , L. Zhao , et al., “A Scalable High‐Porosity Wood for Sound Absorption and Thermal Insulation,” Nature Sustainability 6 (2023): 306.

[21] H. Guan , Z. Cheng , and X. Wang , “Highly Compressible Wood Sponges With a Spring‐Like Lamellar Structure as Effective and Reusable Oil Absorbents,” ACS Nano 12, no. 10 (2018): 10365–10373, 10.1021/acsnano.8b05763. 30272949

[22] J. Ma , J. Li , P. Guo , et al., “Tailoring Microstructures of Carbon Fiber Reinforced Carbon Aerogel‐Like Matrix Composites by Carbonization to Modulate Their Mechanical Properties and Thermal Conductivities,” Carbon 196 (2022): 807.

[23] S. Yang , Z. Wang , Z. Liu , and Y. Wu , “Enhancing Durable Fire Safety and Anti‐Corrosion Performance of Wood Through Controlled in‐Situ Self‐Assembly Synthesis of Ag‐PW Nanospheres,” Chemical Engineering Journal 475 (2023): 145227, 10.1016/j.cej.2023.145227.

[24] B. Liu , X. Lu , H. Meng , G. Pan , and D. Li , “Dispersion of in‐Situ Controllably Grown Nano‐SiO_2_ in Alkaline Environment for Improving Cement Paste,” Construction and Building Materials 369 (2023): 130460.

[25] Q. Ban , Y. Li , L. Li , et al., “Amorphous Carbon Engineering of Hierarchical Carbonaceous Nanocomposites Toward Boosted Dielectric Polarization for Electromagnetic Wave Absorption,” Carbon 202 (2023): 1011–1024.

[26] Y. Ai , R. Xing , R. Huang , J. Kong , and R. Su , “Biomass‐Derived Fire‐Retardant Porous Carbon towards Efficient Electromagnetic Wave Absorption and Shielding,” Carbon 227 (2024): 119268.

[27] M. Qin , L. Zhang , and H. Wu , “Dielectric Loss Mechanism in Electromagnetic Wave Absorbing Materials,” Advanced Science 9, no. 10 (2022): 2105553, 10.1002/advs.202105553. PMC8981909 35128836

[28] J. Kim , M. Hong , D. Lee , et al., “Mechanically Robust Phase‐Change Multiscale‐Architected Metastructures Integrating Asymmetric MXene/T‐CNF Aerogel for Thermal Energy Storage and Electromagnetic Interference Shielding,” Advanced Functional Materials 36, no. 3 (2026): 14180, 10.1002/adfm.202514180.

[29] J. Lee , H. Han , D. Noh , et al., “Multiscale Porous Architecture Consisting of Graphene Aerogels and Metastructures Enabling Robust Thermal and Mechanical Functionalities of Phase Change Materials,” Advanced Functional Materials 34, no. 42 (2024): 2405625, 10.1002/adfm.202405625.

[30] T. Wang , W. Zhao , Y. Miao , et al., “Enhancing Defect‐Induced Dipole Polarization Strategy of SiC@MoO_3_ Nanocomposite towards Electromagnetic Wave Absorption,” Nano‐Micro Letters 16, no. 1 (2024): 273, 10.1007/s40820-024-01478-2. PMC11327238 39147921

[31] J. Calvo‐de la Rosa , A. Bou‐Comas , J. Manel Hernàndez , J. Tejada , and E. M. Chudnovsky , “New Approach to Designing Functional Materials for Stealth Technology: Radar Experiment With Bilayer Absorbers and Optimization of the Reflection Loss,” Advanced Functional Materials 34, no. 6 (2024): 2308819, 10.1002/adfm.202308819.

[32] Y. Liu , S. Zheng , Y. Yang , M. Han , Z. Zeng , and N. Wu , “Advanced Aerogels for High‐Efficiency Electromagnetic Wave Shielding and Absorption,” Advanced Functional Materials 36, no. 37 (2026): 28226, 10.1002/adfm.202528226.

[33] Z. Zeng , H. Jin , M. Chen , W. Li , L. Zhou , and Z. Zhang , “Lightweight and Anisotropic Porous MWCNT/WPU Composites for Ultrahigh Performance Electromagnetic Interference Shielding,” Advanced Functional Materials 26, no. 2 (2016): 303–310, 10.1002/adfm.201503579.

[34] Z. Zeng , C. Wang , G. Siqueira , et al., “Nanocellulose‐MXene Biomimetic Aerogels With Orientation‐Tunable Electromagnetic Interference Shielding Performance,” Advanced Science 7, no. 15 (2020): 2000979, 10.1002/advs.202000979. PMC7404164 32775169

[35] Z. Li , S. Zhang , J. Tao , et al., “Mesoporous Silica@Polypyrrole and Its Epoxy Composites With Ultra‐Broadband Electromagnetic Wave Absorption Properties,” Chemical Engineering Journal 491 (2024): 152051.

[36] Y. Gao , Z. Lei , L. Pan , et al., “Lightweight chitosan‐derived carbon/rGO aerogels loaded With hollow Co1‐Ni O nanocubes for superior electromagnetic wave absorption and heat insulation,” Chemical Engineering Journal 457 (2023): 141325, 10.1016/j.cej.2023.141325.

[37] Z. Zeng , T. Wu , D. Han , Q. Ren , G. Siqueira , and G. Nyström , “Ultralight, Flexible, and Biomimetic Nanocellulose/Silver Nanowire Aerogels for Electromagnetic Interference Shielding,” ACS Nano 14, no. 3 (2020): 2927–2938, 10.1021/acsnano.9b07452. 32109050

[38] Z. Y. Wang , Z. C. Li , B. Li , et al., “Functional Carbon Springs Enabled Dynamic Tunable Microwave Absorption and Thermal Insulation,” Advanced Materials 36, no. 49 (2024): 2412605, 10.1002/adma.202412605. 39428894

[39] L. Yao , W. Yang , S. Zhou , H. Mei , L. Cheng , and L. Zhang , “Design Paradigm for Strong‐Lightweight Perfect Microwave Absorbers: The Case of 3D Printed Gyroid Shellular SiOC‐Based Metamaterials,” Carbon 196 (2022): 961–971, 10.1016/j.carbon.2022.06.004.

[40] Z. Zhao , L. Zhang , and H. Wu , “Hydro/Organo/Ionogels: “Controllable” Electromagnetic Wave Absorbers,” Advanced Materials 34 (2022): 2205376. 10.1002/adma.202205376 36067008

[41] Y. Ai , R. Xing , N. Ren , et al., “Biomass‐Derived Hierarchical Carbon Frameworks Enable Robust Microwave Absorption,” Matter 8 (2025): 102289.

[42] W. Zhan , Y. Hu , Z. Zhao , et al., “BN Nanosheets‐Regulated Elastic Carbon Aerogel Microwave Absorber: Strain‐Driven Broadband Frequency Shifting Over S‐Ku Bands With Sustained Large Bandwidth at High Strains,” Advanced Functional Materials 36, no. 16 (2026): 202519285, 10.1002/adfm.202519285.

[43] L. Liu , S. Zhang , F. Yan , et al., “Three‐Dimensional Hierarchical MoS_2_ Nanosheets/Ultralong N‐Doped Carbon Nanotubes as High‐Performance Electromagnetic Wave Absorbing Material,” ACS Applied Materials & Interfaces 10, no. 16 (2018): 14108–14115, 10.1021/acsami.8b00709. 29616795

[44] J. Wang , L. Liu , S. Jiao , K. Ma , J. Lv , and J. Yang , “Hierarchical Carbon Fiber@MXene@MoS_2_ Core‐sheath Synergistic Microstructure for Tunable and Efficient Microwave Absorption,” Advanced Functional Materials 30, no. 45 (2020): 2002595, 10.1002/adfm.202002595.

[45] F. Yin , H. Lin , W. Wang , et al., “Multifunctional Anisotropic Aerogels for Intelligent Electromagnetic Wave Absorption,” Advanced Functional Materials 35, no. 14 (2024): 2418257, 10.1002/adfm.202418257.

[46] X. Han , Y. Huang , S. Gao , G. Zhang , T. Li , and P. Liu , “A Hierarchical Carbon Fiber@MXene@ZnO Core‐Sheath Synergistic Microstructure for Efficient Microwave Absorption and Photothermal Conversion,” Carbon 183 (2021): 872.

[47] J. Li , Y. Xie , W. Lu , and T. Chou , “Flexible Electromagnetic Wave Absorbing Composite Based on 3D rGO‐CNT‐Fe_3_O_4_ Ternary Films,” Carbon 129 (2018): 76–84, 10.1016/j.carbon.2017.11.094.

[48] P. Wang , L. Cheng , Y. Zhang , and L. Zhang , “Flexible SiC/Si_3_N_4_ Composite Nanofibers With in Situ Embedded Graphite for Highly Efficient Electromagnetic Wave Absorption,” ACS Applied Materials & Interfaces 9 (2017): 28844. 10.1021/acsami.7b05382 28799331

[49] D. Yang , S. Dong , T. Cui , et al., “Multifunctional Carbon Fiber Reinforced C/SiOC Aerogel Composites for Efficient Electromagnetic Wave Absorption, Thermal Insulation, and Flame Retardancy,” Small 20 (2024): 2308145. 10.1002/smll.202308145 38150646

[50] Z. Zhao , M. Yang , L. Li , et al., “A Robust Flexible‐Flexible Synergistic Superhydrophobic Structure for Deicing/Long Icing Delay and Microwave Absorption,” Chemical Engineering Journal 510 (2025): 161654.

[51] M. Yan , Y. Pan , P. He , L. Gong , Y. Fu , and X. Cheng , “Hyperelastic and Multifunctional SiC/SiO_2_ Composite Aerogels With Excellent Mechanical, Thermal Insulation and Electromagnetic Wave Absorbing Properties,” Composites Part A: Applied Science and Manufacturing 186 (2024): 108408, 10.1016/j.compositesa.2024.108408.

[52] D. Wang , M. Zhang , Y. Guo , et al., “Facile Preparation of a Cellulose Derived Carbon/BN Composite Aerogel for Superior Electromagnetic Wave Absorption,” Journal of Materials Chemistry C 10, no. 13 (2022): 5311–5320, 10.1039/D2TC00205A.

[53] H. Wang , X. Sun , S. Yang , et al., “3D Ultralight Hollow NiCo Compound@MXene Composites for Tunable and High‐Efficient Microwave Absorption,” Nano‐Micro Letters 13, no. 1 (2021): 206, 10.1007/s40820-021-00727-y. PMC8505608 34633551

[54] J. Zhou , W. Liu , M. Han , et al., “Machine Learning‐Guided Additive Manufacturing of Multilayer Aerogels for Ultrabroadband, Ultralow‐Reflection Electromagnetic Shielding,” Advanced Materials (2026): 74565, 10.1002/adma.74565. 42571615

[55] Y. Liu , N. Wu , Q. Zhao , et al., “Continuous‐Conductivity‐Gradient All‐Organic Aerogels With Machine‐Learning‐Assisted Design Toward Ultrabroadband, Ultralow‐Reflection Electromagnetic Shielding,” Advanced Materials (2026): 74414, 10.1002/adma.74414. 42541733

[56] Y. Sui , N. Wu , Y. Liu , et al., “Hydrophobic Interaction‐Directed Solvent‐Free Ambient‐Pressure‐Dried MXene Aerogels,” Advanced Materials 38, no. 11 (2026): 14667, 10.1002/adma.202514667. 41504550

[57] G. Shao , D. A. H. Hanaor , X. Shen , and A. Gurlo , “Freeze Casting: From Low‐Dimensional Building Blocks to Aligned Porous Structures—A Review of Novel Materials, Methods, and Applications,” Advanced Materials 32, no. 17 (2020): 1907176, 10.1002/adma.201907176. 32163660

[58] X. Guo , Y. Liu , G. Yao , et al., “Perspective of Chitosan‐Based Electromagnetic Interference Shielding Materials,” Journal of Materials Science and Technology 281 (2026): 21–33.

[59] J. Lin , J. Zhou , G. Yao , et al., “Lightweight Porous Electromagnetic Interference Shielding Materials,” Journal of Materials Science and Technology 283 (2026): 24–38.

[60] L. Chen , Y. Rong , X. Zhou , et al., “MOF‐Derived Carbon Composites: Magnetically Induced High Electromagnetic Shielding Effectiveness and Intelligent Regulation of Absorption‐Dominated Mechanism,” Chemical Engineering Journal 541 (2026): 177744, 10.1016/j.cej.2026.177744.

[61] H. Zhu , Y. Hu , X. Men , et al., “Design of Cf/SiCf/Si_3_N_4_f Multifiber Layered Composite With Enhanced Electromagnetic Wave Absorption Properties,” Journal of the American Ceramic Society 108, no. 4 (2025): 20301, 10.1111/jace.20301.

[62] X. An , Z. Sun , J. Shen , et al., “Heterogeneous Interface Enhanced Polyurethane/MXene@Fe_3_O_4_ Composite Elastomers for Electromagnetic Wave Absorption and Thermal Conduction,” International Journal of Minerals, Metallurgy and Materials 32, no. 3 (2025): 728–737, 10.1007/s12613-024-3025-2.

[63] V. Drach , M. Wiener , G. Reichenauer , H. P. Ebert , and J. Fricke , “Determination of the Anisotropic Thermal Conductivity of a Carbon Aerogel–Fiber Composite by a Non‐Contact Thermographic Technique,” International Journal of Thermophysics 28, no. 5 (2007): 1542–1562, 10.1007/s10765-006-0145-z.

[64] C. Ye , Z. An , and R. Zhang , “Super‐Elastic Carbon‐Bonded Carbon Fibre Composites Impregnated with Carbon Aerogel for High‐Temperature Thermal Insulation,” Advances in Applied Ceramics 118, no. 5 (2019): 292–299, 10.1080/17436753.2019.1572341.

