# Supplementary material for: Wood Carbon Sponge/SiO2 Aerogel Composites with Anisotropic Broadband Microwave Absorption, Elastic Resilience, and Thermal Insulation
Source: Adv Sci (Weinh). 2026 Sep 21:e77888. Online ahead of print. doi: 10.1002/advs.77888 (PMC13592232; doi:10.1002/advs.77888)
Supplement: Supplementary file 1 [file ADVS-9999-e77888-s001.docx]

**Supporting Information**

Wood Carbon Sponge/SiO_2_ Aerogel Composites with Anisotropic Broadband Microwave Absorption, Elastic Resilience, and Thermal Insulation

Shuo Sun†, Zhimeng Zhao, Yonggang Jiang, Fengqi Liu, Yijie Hu*, Junzong Feng, Jian Feng

**Electromagnetic wave (EMW) absorption performance analysis**

The attenuation constant (α) can be calculated by the following formula:

$\alpha=\frac{\sqrt{2}\pi f}{c}\times\sqrt{\left( \mu^{''}\varepsilon^{''}-\mu^{'}\varepsilon^{'} \right)+\sqrt{\left( \mu^{''}\varepsilon^{''}-\mu^{'}\varepsilon^{'} \right)^{2}+\left( \mu^{'}\varepsilon^{''}+\mu^{'}\varepsilon^{''} \right)^{2}}}$	(S1)

IRC of samples can be calculated by the following formula:

$IRC=\left| {\left[ \left( \mu_{r}/\varepsilon_{r} \right)^{1/2}-1 \right]^{2}}/{\left[ \left( \mu_{r}/\varepsilon_{r} \right)^{1/2}+1 \right]^{2}} \right|$	(S2)

The reflection loss (RL) values are used to represent the EMW absorption intensity of materials, which can be obtained by transmission line theory and calculated by the following formula:

$Z_{in}=Z_{0}\sqrt{\left| {\mu_{r}}/{\varepsilon_{r}} \right|}\tanh\left[ j\left( \frac{2\pi fd}{c} \right)\sqrt{\mu_{r}\varepsilon_{r}} \right]$	（S3）

$RL=20{log}_{10}\left| \frac{z_{in}-z_{0}}{z_{in}+z_{0}} \right|$	（S4）

Where Z_in_ represents the normalized input impedance of the obsorber, Z_0_ is the impedance of free space, μ_r_ is the relative complex permeability (μ_r_= μ'- jμ"), ε_r_ is the complex permittivity (ε_r_= ε'- jε"), d is the thickness of absorber, c and f are velocity and microwave frequency, respectively.

$\varepsilon_{c}^{”}$ and $\varepsilon_{p}^{"}$ are the dielectric loss contributed by charge transport and polarization relaxation, respectively, which can be obtained according to Debye theory (S5 and S6):

$\left( \varepsilon^{'}-\frac{\varepsilon_{s}+\varepsilon_{\infty}}{2} \right)+{(\varepsilon^{"})}^{2}={(\frac{\varepsilon_{s}+\varepsilon_{\infty}}{2})}^{2}$	(S5)

$\varepsilon^{"}=\varepsilon_{c}^{”}+\varepsilon_{p}^{"}=\frac{\sigma}{\omega\varepsilon_{0}}+\frac{\varepsilon_{s}-\varepsilon_{\infty}}{1+\omega^{2}\tau^{2}}\omega\tau$	(S6)

Where $\varepsilon_{s}$ is the relative permittivity at static, $\varepsilon_{\infty}$ is the relative permittivity in the high-frequency limit, τ is the polarization relaxation time, ω is the angular frequency, σ is the conductivity.

**Figure S1.**The optical images of NW, WS and WCS.

**Figure S2.**The density of NW, WS, WCS and WCS-SiO_2_.

**Figure S3.**The SEM images of NW and WS in axial, radial and tangential directions.

**Figure S4**. 3D micron-scale CT analysis of WCS-SiO_2._

**Figure S5**. (a) The N_2_ adsorption-desorption isotherms and (b-c) pore size distributions of WCS and WCS-SiO_2_.

**Figure S6.**(a) XPS survey spectra of NW, WS, WCS, and WCS-SiO_2_, respectively. (b) C 1s and (c) Si 2p high resolution spectra of WCS-SiO_2_.

**Figure S7**. (a) Raman spectra of NW, WS, WCS, and WCS-SiO2, respectively. (b) The I_D_/I_G_ of WCS and WCS-SiO_2_.

**Figure S8**. Schematic illustration of the specimen cutting geometry and the alignment of wood anatomical axes with the electric field and propagation direction using the coaxial ring method for anisotropic permittivity determination.

(i)For the axial permittivity, the ring was cut from the axial cross-section with the wood growth direction aligned with the annulus axis, such that the probing electric field oscillates within the radial-tangential plane of the wood.

(ii)For the radial permittivity, the ring was cut from the radial cross-section with the wood ray direction aligned with the annulus radial direction, so that the electric field oscillates within the axial-tangential plane.

(iii)For the tangential permittivity, the ring was cut from the tangential cross-section with the direction perpendicular to the wood rays aligned with the annulus radial direction, and the electric field oscillates within the axial-radial plane.

For transversely isotropic materials, the measured permittivity is dominated by the dielectric response along the anatomical axis aligned with the radial electric field. For the orthotropic wood carbon sponge, where the radial and tangential permittivities may differ, the two in-plane components contribute to the effective permittivity according to their angular orientation around the annulus circumference. Because the ring width is only 2 mm and the specimen is spatially uniform, the measured scalar permittivity closely approximates the response along the aligned anatomical axis. In this work, the tangential direction specifically refers to measurements obtained from rings excised from the tangential cross-section, providing the in-plane permittivity component of the conductive carbon lamellae. This protocol ensures that the directional ordering reported herein faithfully reflects the intrinsic anisotropy of the wood carbon sponge.

**Figure S9.** Two-dimensional contour RL plots of WCS and WCS-SiO_2_ in tangential, radial, and axial directions, respectively.

**Figure S10.** Comparison of RL and EAB between WCS and WCS-SiO_2_ at identical thicknesses along the radial and axial directions.

**Figure S11.** Comparison of theoretical quarter-wavelength thickness and actual optimized thickness for RL_min_ peaks of WCS-SiO_2_-R.

**Figure S12.** The RCS resluts of WCS-SiO_2_-A at 8.05 mm and 5.35 GHz.

**Figure S13.** The RCS resluts of WCS-SiO_2_-R at 2.97 mm and 18 GHz.

**Figure S14**. The time dependence of the surface temperature of the cold side of WCS-SiO_2_ in tangential, radial, and axial directions at hot plate of 150 ℃.

、

**Figure S15**. Compression diagrams of WCS and WCS-SiO_2_ in tangential, radial, and axial directions.

**Figure S16**. Residual strain and Energy dissipation of WCS-SiO_2_ in tangential directions.

**Table S1** Atomic percentages of C, O and Si of NW, WS, WCS and WCS-SiO_2_ from XPS survey spectra.

| Sample | C (at%) | O (at%) | Si (at%) |
| --- | --- | --- | --- |
| NW | 70.32 | 29.68 | / |
| WS | 60.18 | 39.82 | / |
| WCS | 90.64 | 9.36 | / |
| WCS-SiO_2_ | 39.47 | 37.5 | 23.03 |

**Table S2** Parameters of paraffin infiltration for WCS and WCS-SiO_2_.

|  | WCS | WCS-SiO_2_ |
| --- | --- | --- |
| Density before impregnation (g/cm^3^) | 0.031±0.003 | 0.151±0.012 |
| Density after impregnation (g/cm^3^) | 0.824±0.04 | 0.899±0.015 |
| Paraffin mass fraction (wt%) | 94.5±2.2% | 70.6±2.0% |
| Paraffin volume fraction (vol%) | 95.1±2.5% | 40±1.4% |

The paraffin mass fraction was determined as (mass gain / mass after infiltration) × 100%. The paraffin volume fraction was estimated using the paraffin density of ~0.9 g/cm^3^.

**Table S3** Detailed Comparison of Electromagnetic Wave Absorption Performance for this work and Other Reported Materials

| Material Name | No. | RL_min_  (dB) | EAB_max_  (GHz) | Ref. |
| --- | --- | --- | --- | --- |
| WCS-R | ★ | -24.5 (2.91 mm) | 9.7 (4.09 mm) | This work |
| WCS-A | ★ | -66.6 (7.37 mm) | 8.5 (10 mm) | This work |
| WCS-SiO_2_-T | ★ | -64.1 (3.15 mm) | 9.3 (4.09 mm) | This work |
| WCS-SiO_2_-R | ★ | -22.8 (2.97 mm) | 11.6 (4.95 mm) | This work |
| WCS-SiO_2_-A | ★ | -61.9 (8.05 mm) | 8.0 (9.32 mm) | This work |
| CCB-3 | 1 | -60.09 (2.8 mm) | 8.74 (3.6 mm) | [1] |
| MoS_2_ NS/U-NCNTs | 2 | -38.30 (3.5 mm) | 5.40 (2.5 mm) | [2] |
| RCP aerogel | 3 | -61.5 (8.0 mm) | 6.60 (8.0 mm) | [3] |
| CF@MXene@MoS_2_ | 4 | -61.51 (3.5 mm) | 7.60 (2.1 mm) | [4] |
| CF@ MXene@ ZnO | 5 | -67.35 (3.5 mm) | 5.44 (4.0 mm) | [5] |
| CNT film-Fe_3_O_4_  -rGO-PDMS | 6 | -50.50 (5.68 mm) | 5.7 (5.68 mm) | [6] |
| SiC/Si_3_N_4_ | 7 | -57.8 (1.9 mm) | 6.4 (2.5 mm) | [7] |
| CF/CS composite | 8 | -65.45 (2.91 mm) | 4.92 (1.9 mm) | [8] |
| FFSSS | 9 | -51.67 (2.26 mm) | 8.56 (2.26 mm) | [9] |
| SiC/SiO_2_ composite aerogel | 10 | -31.60 (2.0 mm) | 6.50 (2.4 mm) | [10] |
| CCA/BN | 11 | -49.20 (3.75 mm) | 8.16 (3.45 mm) | [11] |
| NiCo@MXene | 12 | -67.22 (1.7 mm) | 6.72 (2.1 mm) | [12] |
| PCW-PCF | 13 | -65.8 (4.6 mm) | 8.35 (4.6 mm) | [13] |

**Supplemental References**

[1] W. Zhan, Y. Hu, Z. Zhao, Y. Peng, J. Chen, L. Li, Y. Jiang, M. Yang, J. Feng, J. Feng, *Adv. Funct. Mater*. **2026**, 36, 202519285.
[2] L. Liu, S. Zhang, F. Yan, C. Li, C. Zhu, X. Zhang, Y. Chen, *ACS Appl.Mater. Interfaces* **2018**, 10, 14108.
[3] F. Yin, H. Lin, W. Wang, L. Zhao, R. Paul, J. Li, W. Liu, D. Kong, Y. Jiang, C. Hu, *Adv. Funct. Mater*. **2024**, 35, 2418257
[4] J. Wang, L. Liu, S. Jiao, K. Ma, J. Lv, J. Yang, *Adv. Funct. Mater.* **2020**,30, 2002595.
[5] X. Han, Y. Huang, S. Gao, G. Zhang, T. Li, P. Liu, *Carbon* **2021**, 183, 872.
[6] J. Li, Y. Xie, W. Lu, T. Chou, *Carbon* **2018**,129, 76.
[7] P. Wang, L. Cheng, Y. Zhang, L. Zhang, *ACS Appl. Mater. Interface*s **2017**, 9, 28844.
[8] D. Yang, S. Dong, T. Cui, J. Xin, X. Xu, J. Chen, Y. Xie, G. Chen, C. Hong, X. Zhang, *Small* **2024**, 20, 2308145.
[9] Z. Zhao, M. Yang, L. Li, Y. Jiang, H. Liu, Y. Hu, J. Feng, J. Feng, *Chem. Eng. J*. **2025**, 510, 161654.
[10] M. Yan, Y. Pan, P. He, L. Gong, Y. Fu, X. Cheng, *Compo. Part A-Appl. Sci. Manuf*. **2024**, 186, 108408.
[11] D. Wang, M. Zhang, Y. Guo, T. Bai, H. Liu, C. Liua, C. Shen, *J. Mater. Chem. C* **2022**, 10, 5311.
[12] H. Wang, X. Sun, S. Yang, P. Zhao, X. Zhang, G. Wang, Y. Huang, *Nanomicro Lett*. **2021**, 13, 206.
[13] Y. Ai, R. Xing, N. Ren, R. Huang, M. Cui, R. Su, J. Kong, *Matter* **2025**, 8, 102289.
